# Case Report: Transient monocular vision loss with isolated paracentral acute middle maculopathy on optical coherence tomography: beware of giant cell arteritis!

**DOI:** 10.3389/fneur.2025.1672043

**Published:** 2025-10-01

**Authors:** George Alencastro Landim, Etienne Bénard-Séguin, Nancy J. Newman, Valérie Biousse

**Affiliations:** ^1^Department of Ophthalmology, Emory University School of Medicine, Atlanta, GA, United States; ^2^Department of Ophthalmology, Cumming School of Medicine, University of Calgary, AB, Canada; ^3^Department of Neurology, Emory University School of Medicine, Atlanta, GA, United States; ^4^Department of Neurological Surgery, Emory University School of Medicine, Atlanta, GA, United States

**Keywords:** paracentral acute middle maculopathy (PAMM), giant cell arteritis, optical coherence tomography, transient monocular vision loss, retinal arterial ischemia

## Abstract

**Introduction:**

We describe a case of transient monocular vision loss (TMVL) and paracentral acute middle maculopathy (PAMM) on optical coherence tomography (OCT) as the only ocular presentation of biopsy-proven GCA.

**Case description:**

An 80-year-old woman presented 4 days after an episode of TMVL in the right eye, which lasted for 2 hours and spontaneously resolved. She also had jaw claudication for 1 month. Visual acuity was 20/20 in both eyes, with no relative afferent pupillary defect. Funduscopic examination was normal. Humphrey visual fields (HVF 24-2) were full in both eyes. Spectral domain OCT of the right eye demonstrated a focal lesion with increased hyperreflectivity at the level of the inner nuclear layer, consistent with PAMM. Fluorescein angiography and indocyanine angiography were normal. She was immediately treated with intravenous steroids for presumed giant cell arteritis (GCA), confirmed subsequently by temporal artery biopsy.

**Conclusion:**

Most reported GCA patients with PAMM have had permanent vision loss and other obvious funduscopic findings. This unique patient had only TMVL and a normal ophthalmologic examination, including full HVF, and no ischemia on retinal angiographic studies. Immediate macular OCT revealing PAMM in TMVL patients older than age 50 years should suggest GCA and prompt immediate treatment to prevent permanent vision loss.

## Introduction

The most dreaded complication of giant cell arteritis (GCA) is permanent vision loss, most often from anterior ischemic optic neuropathy (AION), not infrequently preceded by episodes of transient monocular vision loss (TMVL) (1). Prompt treatment of GCA patients with TMVL with high-dose intravenous steroids is the only way to prevent permanent vision loss and second eye involvement (1). The presence of systemic symptoms suggestive of GCA and findings of elevated erythrocyte sedimentation rate (ESR), C-reactive protein (CRP) and thrombocytosis are very useful for the diagnosis, but they are not always present, making the decision whether to immediately treat patients with vision complaints with steroids often challenging (1). Recent reports of paracentral acute middle maculopathy (PAMM) on macular optical coherence tomography (OCT) in GCA patients suggest that this OCT finding may be helpful in the early diagnosis of GCA, especially in patients with AION (2–5). PAMM is an OCT finding defined by the presence of a hyperreflective band at the level of the inner nuclear layer (INL) of the retina that indicates focal retinal infarction caused by globally impaired perfusion through the retinal capillary system (6–8). Lesions can be caused by various local retinal vascular diseases and systemic disorders, and the presence of PAMM in a patient with transient vision loss suggests a definite vascular mechanism (7, 8). Most cases of GCA and PAMM reported in the literature have had permanent vision loss and other funduscopic findings, including cotton wool spots, retinal whitening, extensive retinal ischemia from central retinal artery occlusion (CRAO), cilioretinal artery occlusion or AION (2, 3, 5, 8–15) (Table 1). We describe a case of TMVL and PAMM as the only ocular presentation of biopsy-proven GCA.

**Table 1:** Summary of the reports of paracentral acute middle maculopathy in giant cell arteritis identified in the English literature.

**Authors (year)**	**Gender/age**	**TMVL**	**Systemic symptoms of GCA**	**TAB or other**	**ESR and CRP**	**BCVA at presentation**	**Funduscopic and OCT features in addition to PAMM**
Christenbury et al. (12)	M/82	Yes; Multiple for 2 days	Yes	Negative	ESR ↑ CRP ↑	Count fingers	Normal
Kasimov et al. (11)	F/73	Yes; Multiple for 10 days	Yes	Positive	ESR ↑ CRP ↑	Count fingers	Cotton wool spots, AION, cilioretinal artery occlusion
Pellegrini et al. (13)	F/72	Yes; 3 days prior	Yes	Positive	ESR ↑ CRP ↑	20/25 with paracentral scotoma	Retinal whitening
**Our case**	**80/F**	**Yes; 4 days prior**	**Yes**	**Positive**	**ESR** **↑**	**20/20**	**Isolated PAMM**
Pichi et al. (8)	F/67	No	Yes	Positive	NA	20/150	Cilioretinal artery occlusion
	F/67	No	Yes	Positive	NA	20/200	Cilioretinal artery occlusion
Ahuja et al. (10)	F/75	No	No	Positive	Normal	20/80	Cotton wool spots
	M/58	No	No	Positive	ESR ↑	NA	AION
Narala et al. (9)	M/86	No	No	Positive	ESR ↑ CRP ↑	20/60	Cotton wool spots and retinal whitening
Broyles et al. (3)	F/75	No	No	Positive	ESR ↑ CRP ↑	20/30 with paracentral scotoma	Cotton wool spots
Sodhi et al. (15)	F/75	No	No	Positive	ESR ↑ CRP ↑	20/20 with paracentral scotoma	Subtle retinal whitening
Pellegrini et al. (16)	M/63	No	No	Positive	ESR ↑ CRP ↑	20/25 with paracentral scotomas at presentation, but no light perception 3 weeks later	Isolated PAMM at presentation AION 3 weeks later
Mairot et al. (14)	5M/11F; Mean age 81.6	NA	NA	3/3 positive; 10 MRI positive	NA	Mean of 20/400 (range 20/20 to light perception)	6 patients isolated PAMM 10 patients AION or CRAO
Mairot et al. (2)	F/85	NA	NA	Positive	CRP ↑	worse than 20/2,000	AION
	F/66	NA	NA	Positive	CRP ↑	worse than 20/2,000	AION
	F/69	NA	NA	Positive	CRP ↑	20/300	AION
	F/68	NA	NA	Positive	CRP ↑	20/2,000	AION
Klefter et al. (5)	5F/3M; Mean age 74.5	NA	NA	Positive TAB or PET	NA	NA	AION

The top 4 rows include 4 patients who presented with transient monocular visual loss (TMVL), including our case; the subsequent 6 rows include 8 patients who did not have an episode of TMVL; the bottom 3 rows include 28 patients for whom no detail regarding initial visual symptoms were provided.

TMVL, transient monocular vision loss; TAB, temporal artery biopsy; NA, not available; M, male; F, female; ESR, erythrocyte sedimentation rate; CRP, C-reactive protein; GCA, giant cell arteritis; AION, anterior ischemic optic neuropathy; CRAO, central retinal artery occlusion; ↑, elevated above the normal value; PET, positron emission tomography.

## Case report

**Figure 1:**
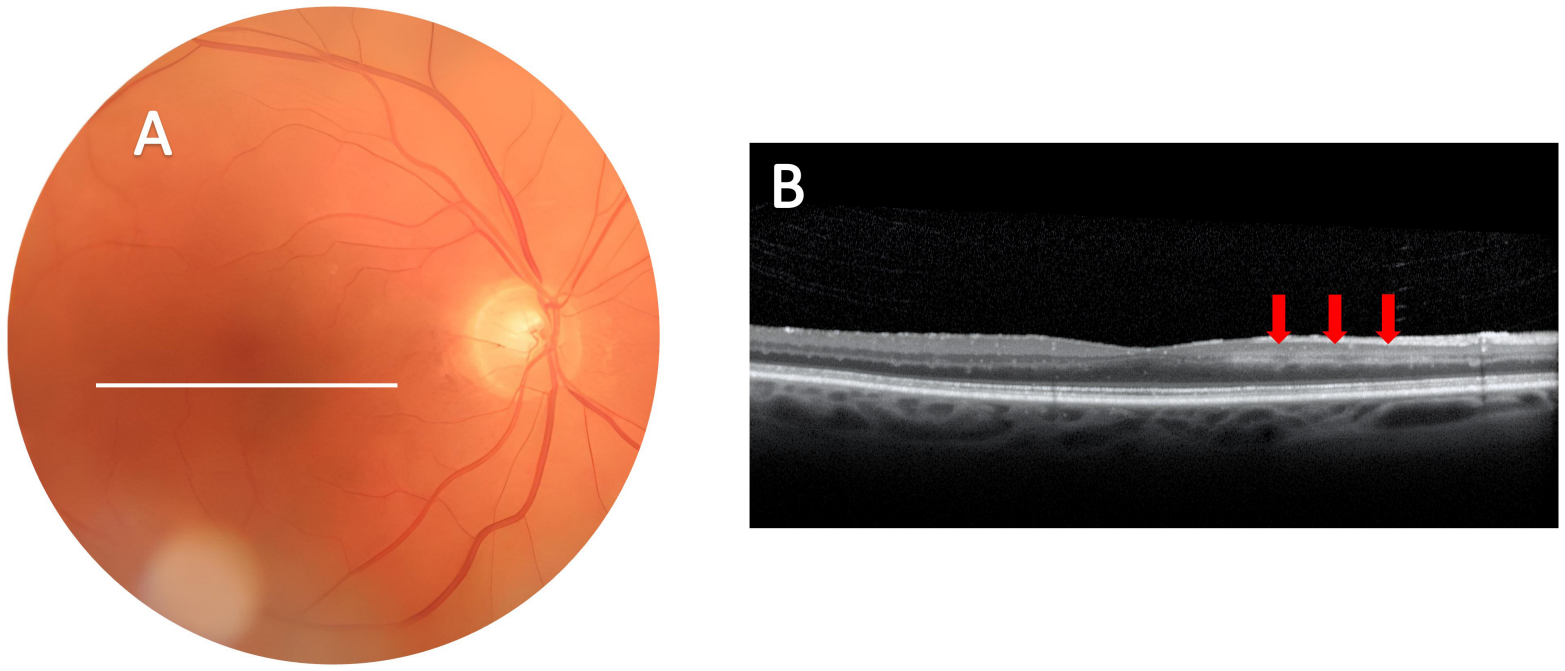
Demonstration of paracentral acute middle maculopathy on ocular imaging obtained four days after transient monocular vision loss in the right eye. **(A)** Color fundus photograph of the right eye is normal. White line corresponds to the scan area of the SD-OCT. **(B)** SD-OCT of the macula shows hyperreflective focal lesions at the level of the inner nuclear layer (red arrows), consistent with ischemia of the deeper vascular complex of the inner retina as seen with PAMM.

**Figure 2:**
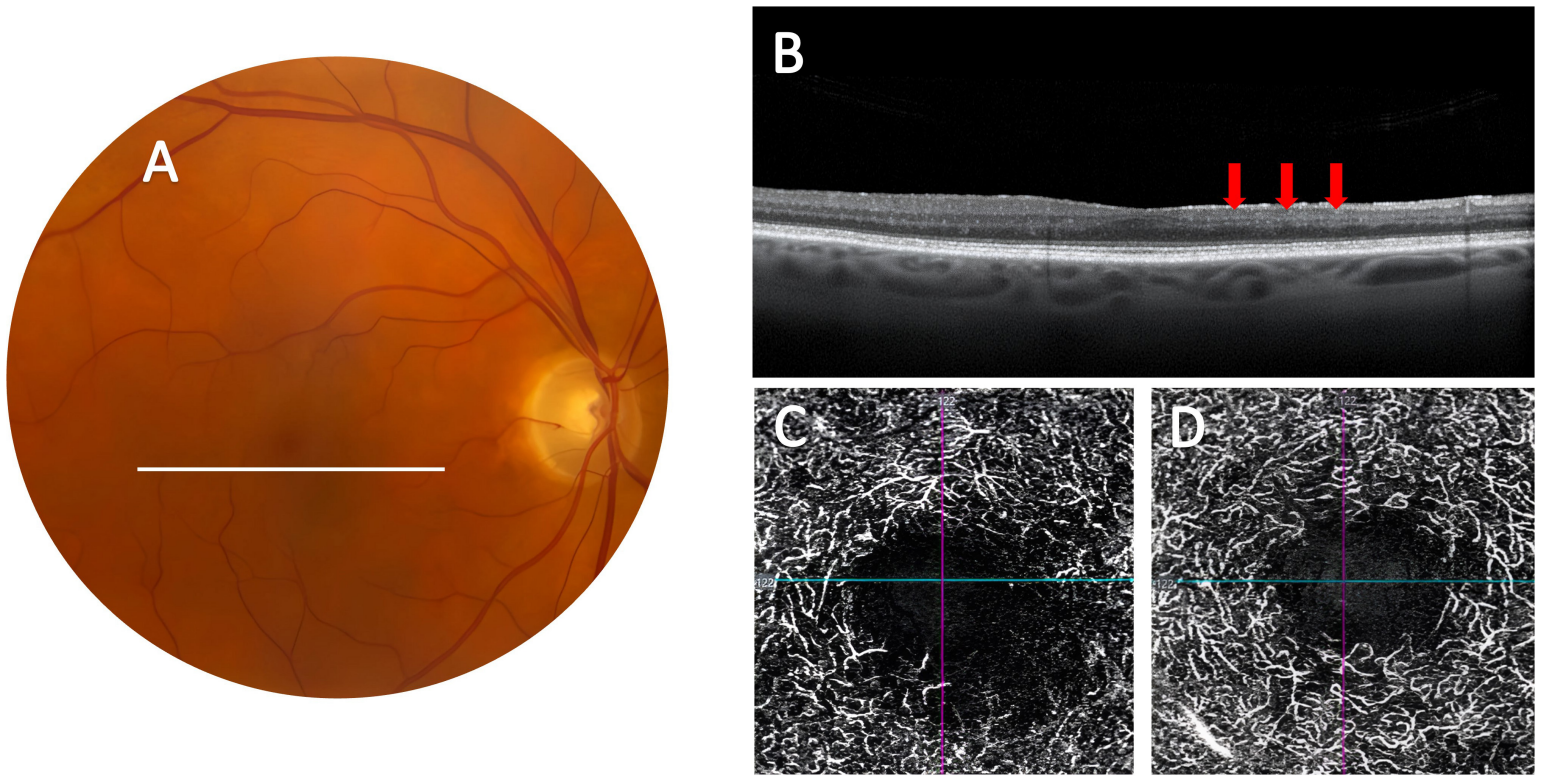
Evolution of paracentral acute middle maculopathy on ocular imaging obtained 36 weeks after presentation. **(A)** Color fundus photograph of the right eye remained normal. **(B)** SD-OCT of the macula shows evolution of the lesion with thinning at the level of the inner nuclear layer (red arrows). **(C)** OCT-A of the DCP with attenuation of the capillaries in the inferonasal parafoveal area the right eye. **(D)** OCT-A with normal parafoveal capillaries of the DCP in the left eye.

An 80-year-old white woman presented to our emergency department (ED) 4 days after an episode of a gray band in the central vision of the right eye that lasted for 2 hours and spontaneously resolved. Medical history was remarkable for polymyalgia rheumatica (PMR) diagnosed 18 months prior, treated with oral prednisone that was discontinued 2 months prior to presentation, and jaw claudication for 1 month. In the ED, the patient had non-mydriatic ocular fundus imaging, including color photographs and OCT of the optic nerve and macula (10). Remote review of these imaging studies showed no obvious retinal or optic nerve abnormalities and no retinal emboli. However, the macular OCT suggested a focal retinal area of hyper-reflectivity in the right macula. The patient was seen immediately by Neuro-ophthalmology where visual acuity was 20/20 in both eyes, color vision was normal and there was no relative afferent pupillary defect. 24-2 SITA-Fast Humphrey visual fields (HVF) were full in both eyes. Funduscopic examination and repeat color fundus photography were normal with no evidence of retinal ischemia. Repeat OCT of the right eye confirmed focal areas of increased hyperreflectivity at the level of the INL, nasal to the macula, consistent with PAMM (Figure 1). Fluorescein angiography (FA) and indocyanine angiography (ICG) were normal, with no choroidal hypoperfusion. ESR was normal at 20 (reference < 30 mm/h) and CRP was mildly elevated at 27 (reference < 10 mg/L). She was admitted to the hospital with high suspicion for TMVL from GCA, and was immediately started on intravenous methyl prednisolone 250 mg, 4 times per day. A right temporal artery biopsy (TAB) performed 1 day after admission demonstrated giant cells in the wall of the artery and around the inner elastic lamina, confirming the diagnosis of GCA. She was discharged 4 days later on oral prednisone and her vision remained normal in both eyes. She was last seen at 36-week follow-up at which time she was taking oral prednisone 5 mg daily and tocilizumab 162 mg injection weekly. She did not have any visual symptoms and repeat 24-2 HVF was normal. Repeat OCT showed an area of atrophy where PAMM was previously noted, correlating with abnormal vasculature on OCT-angiography (OCT-A) (Figure 2).

## Discussion

Our patient's unique presentation of TMVL from GCA with normal visual function and normal funduscopic appearance, but with findings of PAMM on macular OCT 4 days after TMVL, highlights the usefulness of performing a macular OCT acutely in patients with TMVL. Our patient was found to have PAMM on non-mydriatic OCT obtained in our ED (17, 18), which prompted immediate evaluation in our clinic and rapid diagnosis of presumed GCA. She received intravenous steroids rapidly after arriving in the ED, and her visual function remained normal. Although PAMM may be seen in patients with non-arteritic TMVL (19), non-arteritic reperfused CRAO (7, 19) or even central retinal vein occlusions (CRVO) (20), its presence should always suggest the possibility of GCA in patients aged 50 years and older (13–16).

The number of reported cases of PAMM in GCA remains low (Table 1), but this number likely largely underestimates the true prevalence of PAMM in GCA. PAMM is only seen on retinal OCT, and many GCA patients, especially those presenting with vision loss from optic neuropathy or those with isolated TMVL, do not have systematic macular OCT performed acutely. Most reported patients with PAMM and GCA have had permanent vision loss and obvious funduscopic abnormalities suggesting retinal ischemia, such as cotton wool spots, areas of retinal whitening, CRAO or optic disc edema from AION, and were seen in eye clinics where OCT is routinely used. Our patient's ocular fundus was normal, as were retinal FA and ICG, as similarly reported by Sodhi et al. in one patient with PAMM and GCA (15).

PAMM is one of the newer retinal OCT findings that have emerged with the advent of high-resolution spectral domain OCT and OCT-A which provide exquisite images of the retinal layers and retinal vessel changes at various depths (18, 21–24). PAMM is described on OCT as a hyper-reflective band at the level of the retinal INL, eventually leading to permanent thinning of the INL as shown in Figures 1, 2 (7, 8, 22). The INL is located in a region of the retina where oxygen is supplied by both the choroidal and retinal circulations, hence considered a watershed region most susceptible to early ischemia (7, 8, 22–28). The macula's oxygen demand, especially in the inner retinal segments and at the photoreceptor level, is higher than in any other retinal region. Although the choroidal vasculature in the macula is increased to fill this metabolic demand, oxygen diffusion from the choroid to the retina is limited by retinal thickness, which is greatest in the parafoveal region (7, 8, 25), and is often compromised in vasculitic disorders such as GCA (23). PAMM results from focal ischemia of the intermediate and deep capillary plexi, which are responsible for the blood supply to the middle retina which includes the INL (7, 8). This area can be visualized with OCT-A which demonstrates reduced superficial and deep capillary plexi in PAMM (28). We did not perform OCT-A acutely in our patient. However, when seen in follow-up 36 weeks later, OCT-A showed lower capillary density and attenuated deep capillary plexus in the eye with PAMM compared to the fellow eye (Figure 2).

PAMM has been linked to various retinal vascular ischemic disorders, underscoring the critical role of vascular dysfunction in its pathogenesis (7, 8). The literature is mostly based on case reports and small case series, which have reported PAMM in association with CRAO, branch retinal artery occlusions (BRAO), CRVO, Purtscher retinopathy, sickle cell disease, hypercoagulable states, and systemic vasculitic disorders, either auto-immune or post-infectious (7, 8, 26).

PAMM is an important finding in patients with TMVL or those with mild visual symptoms, as selective infarction of the retinal INL may precede devastating subsequent vision loss from severe retinal or optic nerve ischemia (4, 20). Pellegrini et al. reported a 63-year-old man with mild vision loss in one eye who was found to have PAMM as the only finding at initial presentation (16). The diagnosis of GCA was not made at that initial visit and the patient returned 3 weeks later with severe vision loss and pallid optic disc edema in the contralateral eye, consistent with AION from GCA. Among the cases with PAMM and GCA reported in detail in the literature (Table 1), only 3 patients (11–13). other than our case were documented to have experienced TMVL prior to presentation, including only one other patient treated early enough to prevent severe vision loss (13). A recent study suggested that the presence of PAMM in a patient with AION may be a strong argument for GCA, especially in the absence of subretinal fluid tracking from the swollen optic disc toward the macula (5).

## Conclusion

PAMM is a relatively new OCT finding associated with acute retinal ischemia, the significance of which needs to be fully understood before specific recommendations can be made. Our case and other recent reports confirm that PAMM is secondary to focal acute retinal ischemia and may precede more severe retinal ischemia and vision loss. Although most reported patients with GCA and PAMM had other obvious funduscopic findings of ischemia and already had severe vision loss at the time of presentation, the diagnosis of PAMM in a patient with TMVL and normal funduscopic examination should prompt urgent evaluation in order to potentially prevent permanent vision loss from a disorder such as GCA. Similarly, systematic urgent OCT of the macula in patients with TMVL and those with AION may help identify the subgroup of patients at risk for bilateral severe retinal ischemia from GCA. OCT and OCTA are routinely used in eye clinics and are widely available, but may not be systematically performed in patients with isolated TMVL (18). Although very few EDs are equipped with OCTs, the growing interest in non-mydriatic ocular imaging in EDs including with OCT, with remote interpretation by ophthalmologists (17, 18, 29, 30), could greatly improve the evaluation of acute TMVL patients in emergency settings.

## Data Availability

The datasets presented in this article are not readily available because of ethical and privacy restrictions. Requests to access the datasets should be directed to the corresponding author.

## References

[1] Baig I Pascoe A Kini A Lee A. Giant cell arteritis: early diagnosis is key. Eye Brain. (2019) 11:1–12. 10.2147/EB.S170388 30697092 PMC6340646

[2] Mairot K Gascon P Stolowy N Comet A Attia R Beylerian M. Paracentral acute middle maculopathy as a specific sign of arteritic anterior ischemic optic neuropathy. Am J Ophthalmol. 2023; 1:248:1-7. 10.1016/j.ajo.2022.09.019 36228776

[3] Broyles H Chacko J Chancellor J LoRusso F Phillips P Mashayekhi A. Paracentral acute middle maculopathy as the initial presentation of giant cell arteritis. J Neuroophthalmol. (2021) 41:157–9. 10.1097/WNO.0000000000001222 33770010

[4] Bousquet E Santina A Abraham N Daily MJ Sarraf D. Detection of paracentral acute middle maculopathy can prevent blindness and death. Retina. (2023) 43:1827–32. 10.1097/IAE.0000000000003939 37748460

[5] Klefter ON Hansen MS Lykkebirk L Subhi Y Brittain JM Jensen MR. Combining paracentral acute middle maculopathy and peripapillary fluid as biomarkers in anterior ischemic optic neuropathy. Am J Ophthalmol. (2024) 271:329–36. 10.1016/j.ajo.2024.12.001 39645178

[6] Scharf J Freund KB Sadda S Sarraf D. Paracentral acute middle maculopathy and the organization of the retinal capillary plexuses. Prog Retin Eye Res. (2021) 81:100884. 10.1016/j.preteyeres.2020.100884 32783959

[7] Fumi D Ruggeri F Fasciolo D Antonello E Burtini G Abdolrahimzadeh S. Paracentral acute middle maculopathy (PAMM) in ocular vascular diseases-what we know and future perspectives. Vision (Basel). (2025) 9:19. 10.3390/vision9010019 40137931 PMC11946784

[8] Pichi F Fragiotta S Freund KB Au A Lembo A Nucci P. Cilioretinal artery hypoperfusion and its association with paracentral acute middle maculopathy. Br J Ophthalmol. (2019) 103:1137–45. 10.1136/bjophthalmol-2018-312774 30257961

[9] Narala R Reddy SA Mruthyunjaya P. Giant cell arteritis manifesting as retinal arterial occlusion and paracentral acute middle maculopathy in a patient on pembrolizumab for metastatic uveal melanoma. Am J Ophthalmol Case Rep. (2020) 20:100891. 10.1016/j.ajoc.2020.100891 32913923 PMC7472807

[10] Ahuja AS El-Dairi MA Hadziahmetovic M Gospe III SM. Paracentral acute middle maculopathy as a manifestation of giant cell arteritis. J Neuroophthalmol. (2021) 41:153–6. 10.1097/WNO.0000000000001170 33449486

[11] Kasimov M Popovic MM Micieli JA. Paracentral acute middle maculopathy associated with anterior ischemic optic neuropathy and cilioretinal artery occlusion in giant cell arteritis. J Neuroophthalmol. (2022) 42:437–9. 10.1097/WNO.0000000000001306 34238887

[12] Christenbury JG Klufas M Sauer T Sarraf D. OCT angiography of paracentral acute middle maculopathy associated with central retinal artery occlusion and deep capillary ischemia. Ophthalmic Surg Lasers Imaging Retina. (2015) 46:579–81. 10.3928/23258160-20150521-11 26057763

[13] Pellegrini F Mairot K Cuna A Lee AG. Paracentral acute middle maculopathy in giant cell arteritis. Retin Cases Brief Rep. (2024) 18:285–9. 10.1097/ICB.0000000000001381 36730607

[14] Mairot K Sené T Lecler A Philibert M Clavel G Hemmendinger A. Paracentral acute middle maculopathy in giant cell arteritis. Retina. (2022) 42:476–84. 10.1097/IAE.0000000000003339 34723898

[15] Sodhi G Mundae R Lee MS Spencer DB Tang PH. Sudden-onset unilateral painless vision loss. Surv Ophthalmol. (2023) 68:142–5. 10.1016/j.survophthal.2021.10.003 34634290

[16] Pellegrini F Brocca D Calandriello L Cuna A Leonardi F Lee AG. Paracentral acute middle maculopathy as the presenting manifestation of giant cell arteritis. J Neuroophthalmol. (2023) 43:188–91. 10.1097/WNO.0000000000001483 35234683

[17] Berman G Pendley A Wright W Silverman R Kelley C Duran M. Breaking the barriers: methodology of implementation of a non-mydriatic ocular fundus camera in an emergency department. Surv Ophthalmol. (2025) 70:153–61. 10.1016/j.survophthal.2024.09.012 39357747

[18] Biousse V Danesh-Meyer HV Saindane AM Lamirel C Newman NJ. Imaging of the optic nerve: technological advances and future prospects. Lancet Neurol. (2022) 21:1135–50. 10.1016/S1474-4422(22)00173-9 36155662

[19] Betsch D Mishra A. Freund, P. A case of transient monocular vision loss and paracentral acute middle maculopathy. J Neuroophthalmol. (2021) 41:360–2. 10.1097/WNO.0000000000001162 33417419

[20] Louie E Tang A King B. Paracentral acute middle maculopathy presenting as a sign of impending central retinal artery occlusion: a case report. BMC Ophthalmol. (2023) 23:268. 10.1186/s12886-023-02990-6 37312058 PMC10262410

[21] Rahimy E Sarraf D Dollin ML Pitcher JD Ho AC. Paracentral acute middle maculopathy in nonischemic central retinal vein occlusion. Am J Ophthalmol. (2014) 158:372–80. 10.1016/j.ajo.2014.04.024 24794089

[22] Sarraf D Rahimy E Fawzi A Sohn E Barbazetto I Zacks DN. Paracentral acute middle maculopathy a new variant of acute macular neuroretinopathy associated with retinal capillary ischemia. JAMA Ophthalmol. (2013) 131:1275–87. 10.1001/jamaophthalmol.2013.4056 23929382

[23] Pandya U Grinton M Mandelcorn E Felfeli T. Retinal optical coherence tomography imaging biomarkers: a review of the literature. Retina. (2024) 44:369–80. 10.1097/IAE.0000000000003974 37903455 PMC10885864

[24] Yu S Wang F Pang CE Yannuzzi LA Freund B. Multimodal imaging findings in retinal deep capillary ischemia. Retina. (2014) 34:636–46. 10.1097/IAE.0000000000000048 24240565

[25] Abtahi SH Nourinia R Mazloumi M Nouri H Arevalo JF Ahmadieh H. Retinal ischemic cascade: new insights into the pathophysiology and imaging findings. Surv Ophthalmol. (2023) 68:380–7. 10.1016/j.survophthal.2022.11.009 36464134

[26] Rahimy E Kuehlewein L Sadda SR Sarraf D. Paracentral acute middle maculopathy: what we knew then and what we know now. Retina. (2015) 35:1921–30. 10.1097/IAE.0000000000000785 26360227

[27] Moura-Coelho N Gaspar T Ferreira JT.. Paracentral acute middle maculopathy - review of the literature. Graefe's Arch Clin Exp Ophthalmol. (2020) 258:2583–25960. 10.1007/s00417-020-04826-1 32661700

[28] Liu Z Pan X Wang D Zou Y Liu P Wang Y. The clinical features and perfusion density in paracentral acute middle maculopathy by optical coherence tomography angiography study. Photodiagnosis Photodyn Ther. (2024) 50:104380. 10.1016/j.pdpdt.2024.104380 39426652

[29] Bénard-Séguin E Nahab F Pendley A Duran M Torres Soto M Keadey M. Eye stroke protocol in the emergency department. J Stroke Cerebrovasc Dis. (2024) 33:107895. 10.1016/j.jstrokecerebrovasdis.2024.107895 39079617

[30] Lema GM De Leacy R Fara MG Ginsburg RN Barash A Banashefski B. remote consult retinal artery occlusion diagnostic protocol. Ophthalmology. (2024) 131:724–30. 10.1016/j.ophtha.2023.11.031 38349294

